# Multimodal OCT/OCT-A Risk Stratification in Optic Disc Drusen: Drusen Height, Peripapillary Perfusion, and Visual Field Slope Identify Fast Progressors

**DOI:** 10.3390/diagnostics16071024

**Published:** 2026-03-29

**Authors:** Alina Dumitriu, Bogdan Dumitriu, Mihnea Munteanu, Horia Tudor Stanca, Cosmin Rosca

**Affiliations:** 1 Doctoral School, “Victor Babes” University of Medicine and Pharmacy Timisoara, 300041 Timisoara, Romania; alina.moatar@umft.ro (A.D.); bogdan.dumitriu@umft.ro (B.D.); 2 Department of Ophthalmology, “Victor Babes” University of Medicine and Pharmacy Timisoara, 300041 Timisoara, Romania; 3 Ophthalmology Department, “Carol Davila” University of Medicine and Pharmacy, 050474 Bucharest, Romania; horia.stanca@umfcd.ro; 4 Oculens Clinic, Calea Turzii No. 134-136, 400501 Cluj-Napoca, Romania; roscacosmin@yahoo.com

**Keywords:** optic disk drusen, optical coherence tomography angiography, visual fields, retinal nerve fiber layer, disease progression

## Abstract

**Background and Objectives:** Optic disc drusen (ODD) are deposits in the optic nerve head that can look like true swelling, and in some patients, slowly damage the optic nerve and cause visual field loss. We aimed to identify which eyes are most likely to worsen over time using common clinic tests. **Methods:** We studied 131 adults with OCT-confirmed ODD who also had OCT-angiography (a scan that measures small blood vessels around the optic nerve) and repeated visual field tests over at least 18 months. We measured (1) the size of the drusen (maximum drusen height), (2) blood vessel density around and inside the optic nerve, and (3) change in visual field performance over time. “Fast progression” was defined as visual field worsening of ≥0.5 dB per year. **Results:** Eyes with superficial ODD had larger drusen than buried ODD (382.6 ± 110.9 vs. 247.2 ± 92.8 µm; *p* < 0.001) and more frequent visual field defects (78.6% vs. 58.7%; *p* = 0.02). When blood vessel density around the optic nerve was low, fast progression was much more common (52.3%) than in the middle (16.3%) or highest groups (13.6%; *p* < 0.001). In the adjusted model, fast progression was more likely with superficial ODD (OR 6.3) and larger drusen (OR 2.0 per 100 µm), and less likely when the vessel density was higher (OR 0.8 per 1% increase). Adding the vessel measurements improved the prediction accuracy (AUC 0.8 → 0.9; *p* = 0.011). **Conclusions:** Combining drusen size and blood vessel measurements helps identify ODD patients at higher risk of faster visual field loss and may guide closer follow-up.

## 1. Introduction

Optic disc drusen (ODD) are acellular, calcified deposits within the prelaminar optic nerve head that can mimic true disc edema, and in a meaningful subset of patients, behave as a chronic compressive optic neuropathy with progressive visual field loss. Beyond diagnostic confusion (papilledema vs. pseudopapilledema), the practical clinical problem is risk stratification, identifying which eyes are likely to lose function, and therefore require closer longitudinal surveillance and earlier functional testing [1]. Current concepts emphasize that ODD are not simply incidental calcifications, but a dynamic consequence of a crowded optic nerve head architecture in which axoplasmic stasis, local tissue remodeling, and biomechanical stress may interact over time. This framework helps explain why ODD are frequently associated with small, crowded discs, and why functional impairment can evolve even when central acuity remains preserved until late stages [2].

ODD evaluation has shifted toward multimodal imaging, with particular emphasis on optical coherence tomography (OCT). Comparative diagnostic studies show that enhanced-depth imaging OCT (EDI-OCT) has become a high-performing reference technique for detecting ODD, especially when drusen are not clinically visible, supporting its role as a first-line confirmatory test in suspected pseudopapilledema [3]. Earlier work similarly demonstrated that EDI-OCT increases the detection of deeply located (buried) drusen compared with conventional approaches, helping explain why ODD are being recognized more frequently in contemporary neuro-ophthalmic practice [4].

A central reason prognosis is difficult is the marked heterogeneity in ODD morphology and location. EDI-OCT studies comparing eyes with and without visual field loss suggest that morphologic drusen characteristics and associated neuroaxonal thinning differ between phenotypes, implying that the presence of ODD alone is an incomplete descriptor of risk [5]. Quantitative approaches that measure anatomic location and volume further support a structure–risk paradigm in which greater drusen burden and specific topography may relate to greater vulnerability [6]. Newer OCT approaches, including swept-source imaging in selected series, can refine visualization of drusen boundaries and optic nerve head anatomy, further strengthening the mechanistic link between drusen structure and downstream tissue effects [7].

Functionally, standard automated perimetry often reveals enlarged blind spots, arcuate-like defects, nasal steps, or altitudinal patterns with relatively preserved central acuity, an important mismatch that can delay symptom recognition. OCT-derived retinal nerve fiber layer (RNFL) and macular ganglion cell–inner plexiform layer (GCIPL) metrics provide objective markers of neuroaxonal integrity and correlate with visual field status, yet inter-individual variability means similar thickness values can correspond to different functional outcomes [8]. This has motivated interest in complementary biomarkers; OCT angiography (OCT-A) enables the quantification of peripapillary microvasculature, and multimodal studies have linked reduced peripapillary vessel density with more advanced functional loss, supporting a plausible vascular reserve dimension to ODD vulnerability [9,10].

Spatially resolved OCT-A analyses add nuance by demonstrating quadrant-level alterations in peripapillary superficial microvasculature, consistent with topographic coupling between structural stressors and local perfusion [11]. Multimodal studies integrating EDI-OCT-derived drusen segmentation with OCT-A metrics also report inverse relationships between drusen volume/burden and perfusion metrics [12]. Clinical heterogeneity remains substantial—large series have identified multiple factors associated with visual field defects, underscoring the need for models that combine morphology, tissue integrity, and vascular signals rather than relying on any single parameter [13]. Importantly, ODD are not static: longitudinal EDI-OCT work shows that anterior displacement or dynamic change in drusen features can accompany RNFL thinning over time [14]. Longitudinal clinical series also document visual field evolution, highlighting why estimating individualized perimetric slopes may be a practical method to identify fast progressors for closer monitoring [15]. The present study integrates EDI-OCT drusen metrics, OCT-A vessel density, and longitudinal MD slope modeling, testing the hypothesis that microvascular compromise and drusen height together identify fast-progressing ODD.

## 2. Materials and Methods

### 2.1. Study Design, Setting, and Participants

We designed a single-center observational cohort study combining retrospective extraction of standardized imaging/perimetry metrics with the longitudinal modeling of visual field change. The project was developed at the institutions affiliated with the Victor Babes University of Medicine and Pharmacy Timisoara, using a consistent diagnostic pathway and imaging protocols to maximize comparability across patients while introducing novel microvascular endpoints and progression modeling.

Consecutive adult patients evaluated for suspected or confirmed optic disc drusen were eligible if ODD were confirmed by enhanced-depth imaging OCT (EDI-OCT) criteria and if the record contained at least one high-quality OCT-A scan and serial automated perimetry enabling slope estimation. One eye per patient was analyzed to avoid within-subject correlation; the “study eye” was defined as the worse eye based on baseline MD. If both eyes had similar MD, the eye with higher drusen height on EDI-OCT was selected to reflect maximal structural burden.

Inclusion criteria were: age ≥ 18 years, OCT-confirmed ODD, reliable standard automated perimetry with ≥3 tests over ≥18 months, and interpretable OCT-A with acceptable quality indices. Exclusion criteria were designed to reduce confounding optic neuropathies and macular disease: established glaucoma with characteristic cupping progression, advanced diabetic retinopathy, macular degeneration, optic neuritis, compressive optic neuropathy, or neurologic disorders affecting perimetry reliability. Patients with media opacity that prevented acceptable OCT-A signal strength were excluded from OCT-A analyses.

### 2.2. Imaging Acquisition, Classification, and Quantitative Drusen Metrics

All participants underwent spectral-domain OCT with enhanced-depth imaging to confirm the presence of ODD and to quantify structural measures. Global RNFL and global macular GCC thickness were recorded from standardized scan protocols. In addition, drusen morphology was characterized by trained graders using predefined criteria: buried ODD were defined as drusen visible on EDI-OCT without ophthalmoscopically obvious refractile deposits; superficial ODD were defined as deposits clinically apparent and/or clearly anterior on OCT.

We quantified the maximum drusen height (µm) on EDI-OCT as the maximal vertical extent of the largest drusen complex within the optic nerve head on the best-centered B-scan. Measurements were performed using built-in calipers and recorded to one decimal place after standard rounding. Presence of peripapillary hyperreflective ovoid mass-like structures (PHOMS) was recorded as a binary feature. Laterality (unilateral vs. bilateral ODD) was extracted from imaging records.

Because this variable was obtained manually, a standardized caliper-based approach was used on the best-centered EDI-OCT B-scan, and measurements were repeated during image review when drusen borders were indistinct. Formal inter-observer reproducibility statistics were not available in this retrospective dataset and are therefore acknowledged as a limitation.

OCT angiography (OCT-A) was acquired using a disc-centered scan with automated layer segmentation that was manually reviewed for predefined quality-control failures. For the purposes of this study, segmentation failure referred to obvious misidentification or displacement of retinal/optic nerve head boundaries on the corresponding B-scan or en face slab including slab truncation, boundary shifts, or vessel-density maps that were clearly inconsistent with the underlying anatomy. Two quantitative microvascular metrics were recorded: peripapillary vessel density (PeriVD, %) and intra-disc vessel density (DiscVD, %). Scans with motion artifacts, segmentation failure, or insufficient quality (per device index) were excluded. The goal was to use metrics that can be implemented clinically without requiring custom research software.

### 2.3. Functional Testing, Symptoms, and Longitudinal Outcomes

Standard automated perimetry was performed using a 24-2 strategy (or equivalent threshold test) according to routine clinic protocols. Reliability criteria were applied (excessive fixation losses or false positives prompted exclusion of that test). Baseline functional severity was summarized using mean deviation (MD, dB) and pattern standard deviation (PSD, where available). A reproducible visual field defect was recorded when clinic interpretation documented consistent defect patterns across at least two reliable fields.

Patient-reported symptoms were abstracted from structured documentation, focusing on transient visual obscurations (TVOs) and headache history, because these features commonly influence the timing of presentation, the likelihood of neuro-ophthalmic work-up, and follow-up intensity in ODD even when central acuity remains relatively preserved. In this study, symptom abstraction was therefore used to characterize baseline clinical presentation and health-seeking behavior rather than to imply that these symptoms were causal determinants of structural progression.

The longitudinal outcome was MD slope (dB/year) estimated per patient using linear regression of MD over time, anchored to the first reliable baseline test. We defined fast progression as MD slope ≤ −0.5 dB/year to isolate a clinically meaningful subgroup analogous to “fast progressors” in other optic neuropathies. For interpretability, we also summarized 2-year MD deterioration (dB) and 2-year RNFL loss (µm) using modeled change over 24 months (or the closest comparable estimate), emphasizing clinically actionable time windows.

A linear slope was prespecified because the number and spacing of repeat fields varied across patients and linear regression yields an intuitive annualized estimate for routine clinical use. We acknowledge, however, that non-linear trajectories may occur in some eyes and should be evaluated in longer datasets with denser follow-up.

### 2.4. Statistical Analysis

Continuous variables are reported as mean ± SD; categorical variables are reported as *n* (%). Between-group comparisons (buried vs. superficial) used Welch’s *t*-test for continuous outcomes to accommodate unequal variances and χ^2^ tests for categorical outcomes. Fisher’s exact test was used when expected cell counts were <5. For multi-group comparisons across peripapillary vessel density tertiles, we used Welch’s ANOVA (heteroscedasticity-robust). Two-sided *p*-values < 0.05 were considered statistically significant.

Associations between continuous predictors and functional outcomes (baseline MD and MD slope) were evaluated using Pearson correlation coefficients (r) with corresponding *p*-values derived from the t distribution with *n* − 2 degrees of freedom. Correlation direction was interpreted in the clinical sign convention (more negative MD implies worse function; more negative MD slope implies faster decline).

To model independent predictors of fast progression, multivariable logistic regression was constructed with fast progression (yes/no) as the dependent variable. Prespecified predictors included age (per 10 years), drusen height (per 100 µm), peripapillary vessel density (per 1%), baseline MD (per 1 dB), migraine, hypertension, and superficial vs. buried status. Results are reported as adjusted odds ratios (ORs) with 95% confidence intervals (CIs) and *p*-values. Discrimination was summarized using the area under the ROC curve (AUC). Model assumptions (linearity on the logit scale for continuous predictors and absence of extreme multicollinearity) were evaluated pragmatically during analysis.

## 3. Results

Table 1 compares the baseline characteristics between eyes with buried ODD (*n* = 75) and superficial ODD (*n* = 56). The superficial group was older (44.4 ± 11.1 vs. 39.9 ± 13.3 years; *p* = 0.037) and had a higher prevalence of migraine (48.2% vs. 29.3%; *p* = 0.038). Axial length was slightly longer in superficial ODD (22.9 ± 0.9 vs. 22.6 ± 0.8 mm; *p* = 0.033). Sex distribution (female 67.9% vs. 57.3%; *p* = 0.296), arterial hypertension (30.4% vs. 32.0%; *p* = 0.992), hyperlipidemia (42.9% vs. 49.3%; *p* = 0.588), obstructive sleep apnea (14.3% vs. 13.3%; *p* = 1), smoking (21.4% vs. 26.7%; *p* = 0.627), and spherical equivalent (0.9 ± 1.7 vs. 1.1 ± 1.6 D; *p* = 0.475) did not differ significantly between drusen phenotypes.

Table 2 shows that superficial ODD had markedly greater maximum drusen height than buried ODD (382.6 ± 110.9 vs. 247.2 ± 92.8 µm; *p* < 0.001). Intra-disc vessel density was lower in superficial ODD (43.3 ± 2.3% vs. 44.4 ± 2.8%; *p* = 0.019), while peripapillary vessel density did not differ (50.0 ± 2.7% vs. 50.6 ± 2.6%; *p* = 0.255). A reproducible visual field defect was more frequent in superficial ODD (78.6% vs. 58.7%; *p* = 0.02). PHOMS prevalence (58.9% vs. 65.3%; *p* = 0.564), bilateral ODD (58.9% vs. 62.7%; *p* = 0.786), and structural thickness metrics—global RNFL (96.4 ± 11.9 vs. 99.9 ± 9.4 µm; *p* = 0.077) and global GCC (91.4 ± 7.9 vs. 93.0 ± 7.2 µm; *p* = 0.216)—were similar across groups.

Table 3 demonstrates a graded structure–vascular–function relationship across the peripapillary vessel density (VD) tertiles (low 47.3 ± 1.7%, mid 50.3 ± 0.6%, high 52.9 ± 1.4%; *p* < 0.001). Lower VD was associated with thinner neuroaxonal layers (RNFL 93.4 ± 10.0 vs. 99.3 ± 9.7 vs. 100.4 ± 11.9 µm; *p* = 0.005 and GCC 90.6 ± 5.6 vs. 92.4 ± 8.5 vs. 94.1 ± 7.4 µm; *p* = 0.043), reduced contrast sensitivity (logCS 1.4 ± 0.2 vs. 1.6 ± 0.2 vs. 1.6 ± 0.2; *p* < 0.001), greater maximum drusen height (333.4 ± 127.9 vs. 285.6 ± 113.6 vs. 252.9 ± 114.2 µm; *p* = 0.01), and faster functional decline (MD slope −0.4 ± 0.2 vs. −0.3 ± 0.2 vs. −0.2 ± 0.2 dB/year; *p* < 0.001). Consistently, fast progression (≤−0.5 dB/year) was far more common in the low-VD tertile (52.3%) than mid (16.3%) or high VD (13.6%; *p* < 0.001). Baseline VF MD trended worse with lower VD (−4.9 ± 2.5 vs. −4.1 ± 2.5 vs. −3.8 ± 2.8 dB; *p* = 0.093), and transient visual obscurations differed across tertiles (31.8% vs. 58.1% vs. 36.4%; *p* = 0.03).

Table 4 contrasts slow/stable eyes (*n* = 95) with fast progressors (*n* = 36) and shows that fast progression clustered with an adverse structural and microvascular phenotype. Fast progressors were older (47.1 ± 11.8 vs. 39.9 ± 12.3 years; *p* = 0.003) and far more likely to have superficial ODD (75.0% vs. 30.5%; *p* < 0.001). They also had lower peripapillary vessel density (48.8 ± 2.8% vs. 50.7 ± 2.4%; *p* < 0.001), greater maximum drusen height (375.6 ± 114.4 vs. 258.5 ± 109.9 µm; *p* < 0.001), thinner RNFL (93.4 ± 11.9 vs. 99.3 ± 10.1 µm; *p* = 0.01), worse contrast sensitivity (logCS 1.4 ± 0.2 vs. 1.6 ± 0.2; *p* < 0.001), and more impaired baseline VF (MD −5.1 ± 2.1 vs. −3.9 ± 2.8 dB; *p* = 0.009). Over 2 years, fast progressors showed a substantially steeper MD slope (−0.6 ± 0.1 vs. −0.2 ± 0.2 dB/year; *p* < 0.001), greater RNFL loss (2.4 ± 0.8 vs. 1.6 ± 0.9 µm; *p* < 0.001), and larger MD deterioration (1.2 ± 0.4 vs. 0.6 ± 0.5 dB; *p* < 0.001), with VF defects also more frequent (83.3% vs. 61.1%; *p* = 0.027).

Table 5 summarizes the Pearson correlations linking both baseline VF status (MD) and progression rate (MD slope) to structural and vascular measures. Worse baseline MD correlated with greater drusen height (r = −0.2; *p* = 0.026), older age (r = −0.3; *p* < 0.001), and lower contrast sensitivity (logCS r = 0.3; *p* < 0.001), while higher peripapillary vessel density (PeriVD) and thicker GCC showed modest positive associations with better baseline MD (PeriVD r = 0.2; *p* = 0.012; GCC r = 0.2; *p* = 0.013). For progression, faster decline (more negative MD slope) was strongly associated with greater drusen height (r = −0.5; *p* < 0.001) and was also related to lower PeriVD (r = 0.4; *p* < 0.001), lower DiscVD (r = 0.3; *p* = 0.003), thinner RNFL (r = 0.3; *p* < 0.001) and GCC (r = 0.3; *p* = 0.001), older age (r = −0.3; *p* = 0.001), and poorer contrast sensitivity (logCS r = 0.4; *p* < 0.001). BCVA showed a small but significant association with MD slope (r = −0.2; *p* = 0.014), whereas its correlation with baseline MD did not reach significance (r = −0.2; *p* = 0.063).

Table 6 presents the adjusted predictors of fast progression. Greater drusen height independently increased the odds of fast progression (adjusted OR 2.0 per 100 µm; 95% CI 1.3–3.2; *p* = 0.003), while higher peripapillary vessel density was protective (adjusted OR 0.8 per 1% increase; 95% CI 0.6–1.0; *p* = 0.046). Superficial (vs. buried) ODD was the strongest categorical predictor (adjusted OR 6.3; 95% CI 2.1–18.5; *p* < 0.001). Age (per 10 years), baseline MD (per 1 dB), migraine, and hypertension were not statistically significant in the adjusted model (all *p* > 0.18), suggesting that structural burden and microvascular compromise drive progression risk beyond these clinical factors in this cohort.

Table 7 evaluates whether peripapillary VD partially mediates the association between drusen height and 2-year VF worsening (MD deterioration). Per +100.0 µm increase in drusen height, the total effect on 2-year MD deterioration was 0.3 dB (95% CI 0.2 to 0.5; *p* < 0.001). The natural direct effect (not via VD) remained significant at 0.2 dB (95% CI 0.1 to 0.4; *p* = 0.002), while the natural indirect effect through VD was 0.1 dB (95% CI 0.0 to 0.2; *p* = 0.017), corresponding to a proportion mediated of 27.8% (95% CI 11.6 to 46.9; *p* = 0.004). These findings indicate that reduced peripapillary perfusion explains a meaningful, but incomplete, component of the drusen height-related functional decline over 2 years.

Table 8 shows that adding OCT-A vascular metrics improved the prediction of fast progression beyond a clinical + OCT model. Model discrimination increased from AUC 0.8 to 0.9 (Δ = 0.1; *p* = 0.011), overall accuracy improved with a lower Brier score (0.2 to 0.1; Δ = −0.1; *p* = 0.019), and calibration slope modestly improved (0.9 to 1.0; Δ = 0.1; *p* = 0.044), while calibration-in-the-large improved numerically but was not significant (0.1 to 0.0; *p* = 0.062). Reclassification metrics supported added clinical utility: categorical NRI was 22.6% (*p* = 0.008) and IDI was 6.4% (*p* = 0.014), consistent with better separation of fast progressors from slow/stable eyes; the likelihood ratio test also favored the expanded model (*p* = 0.003).

**Figure 1:**
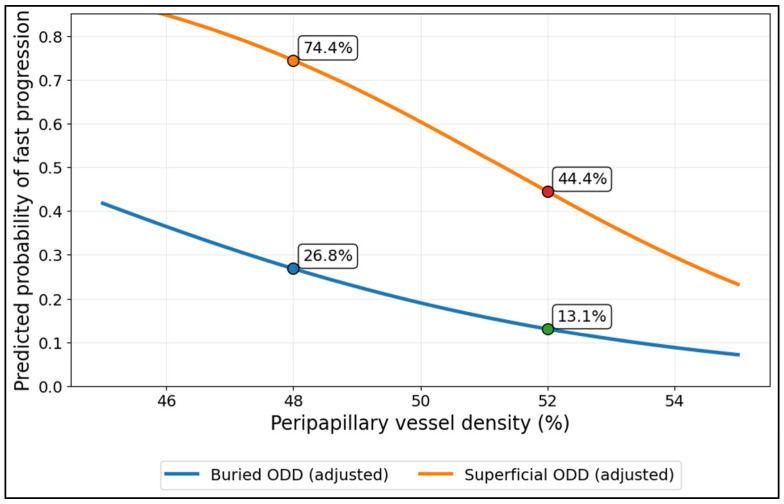
Adjusted probability of fast progression vs. peripapillary vessel density (VD), stratified by buried vs. superficial ODD.

Figure 1 shows a strong VD gradient for fast progression risk, with consistently higher predicted risk in superficial ODD across the same VD range. At VD = 48.0%, the adjusted probability was 26.8% for buried ODD versus 74.4% for superficial ODD. At VD = 52.0%, risk fell to 13.1% (buried) and 44.4% (superficial). The separation between curves suggests that under this model specification, superficial ODD remain higher-risk even when VD is relatively preserved, while increasing VD still confers a protective effect in both groups.

**Figure 2:**
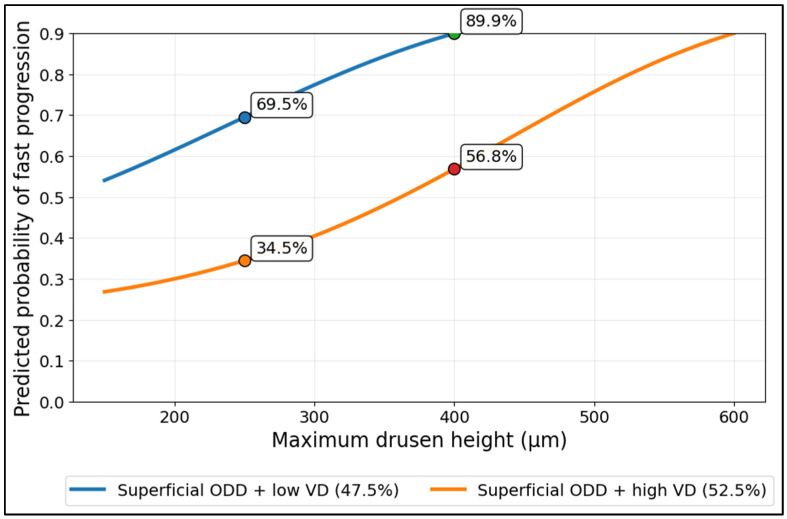
Non-linear drusen-height risk curve in superficial ODD, contrasted at low vs. high peripapillary VD.

At 250 µm, predicted risk was 69.5% under low VD (47.5%) versus 34.5% under high VD (52.5%). At 400 µm, the separation persisted and widened: 89.9% (low VD) versus 56.8% (high VD). Overall, higher VD shifts the entire risk curve downward, indicating a buffering effect of peripapillary perfusion on height-related vulnerability, especially as drusen height increases into higher ranges (Figure 2).

**Figure 3:**
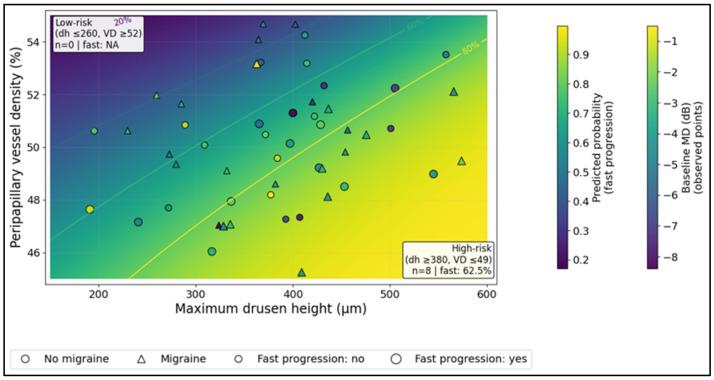
Bivariate risk landscape in superficial ODD: predicted risk surface with observed overlays (baseline MD, migraine, progression status).

In the observed overlays, the high-risk region (defined as drusen height ≥ 380 µm and VD ≤ 49.0%) included *n* = 8 superficial-ODD eyes with an observed fast progression rate of 62.5% (Figure 3).

## 4. Discussion

### 4.1. Analysis of Findings

The present cohort highlights a clinically useful “structure–perfusion–slope” framework for optic disc drusen (ODD) risk stratification. Superficial ODD eyes were older and demonstrated substantially greater drusen height than buried ODD, alongside a higher prevalence of reproducible visual field (VF) defects (78.6% vs. 58.7%) and slightly lower intra-disc vessel density. Beyond phenotype labels, stratification by peripapillary vessel density (VD) revealed a coherent gradient in neuroaxonal integrity and function: lower VD was associated with thinner RNFL/GCC, worse contrast sensitivity, more negative MD slopes, and a markedly higher proportion of fast progressors (52.3% in the lowest VD tertile vs. 13.6% in the highest). Importantly, in multivariable modeling, superficial ODD (adjusted OR 6.3) and drusen height (adjusted OR 2.0 per 100 µm) remained strong predictors of fast progression, while peripapillary VD conveyed independent protective information (adjusted OR 0.8 per 1% higher VD). These findings collectively support a model in which anatomic drusen burden and microvascular reserve jointly shape the trajectory of functional decline.

Our observation that superficial ODD is enriched among fast progressors (75% superficial in fast progressors vs. 30.5% in slow/stable) is consistent with clinical series showing that VF defects are common in ODD and can present with diverse patterns (e.g., enlarged blind spot, arcuate-like or nasal defects), with variability across eyes even within the same patient. In a recent clinical report, VF abnormalities were frequent among patients with optic nerve head drusen, reinforcing that functional impairment is not restricted to a single “advanced” phenotype and may be underrecognized when central acuity remains preserved [16]. Complementing this, Wandji and colleagues specifically emphasized that buried ODD can still exhibit meaningful VF loss and RNFL damage, supporting the notion that “buried” does not equate to benign—particularly when structural compression and axonal compromise are already present [17]. In this context, the higher VF-defect prevalence we observed in superficial ODD likely reflects both greater drusen prominence (and hence mechanical crowding) and the cumulative effect of time, but the substantial defect rate in buried ODD underscores the need for systematic perimetry and OCT-based tissue assessment across phenotypes.

The microvascular signal in our dataset aligns with—and extends—the prior OCT-A literature by linking perfusion metrics directly to future functional slopes. Engelke et al. reported that OCT-A measures in ODD relate meaningfully to structural and functional parameters, supporting the premise that reduced microvascular density accompanies (and potentially compounds) neuroaxonal compromise [18]. Similarly, Cennamo et al. demonstrated OCT-A-detectable flow impairment in optic nerve drusen, providing early evidence that vascular metrics differentiate ODD eyes and may have clinical utility beyond morphology alone [19]. Case-level OCT-A observations further illustrate focal vessel density reductions colocalizing with drusen burden and associated functional symptoms (e.g., contrast sensitivity loss), suggesting that drusen-associated perfusion changes can be spatially specific rather than purely global [20]. Building on these cross-sectional signals, our results add a pragmatic prognostic dimension: lower baseline peripapillary VD was associated with steeper MD decline and meaningfully improved discrimination/calibration when added to a clinical + OCT model, implying that OCT-A contributes incremental, clinically actionable risk information rather than simply duplicating what RNFL thickness already captures.

The inverse association between PeriVD and progression can also be interpreted within a vascular reserve framework, whereby higher baseline peripapillary perfusion may reflect greater capacity of the optic nerve head to tolerate chronic compressive crowding from drusen [18,19,20,21]. At the same time, PeriVD is unlikely to be an isolated local variable. Systemic blood pressure, ocular perfusion pressure, migraine biology, and broader vascular autoregulatory factors may all modulate the OCT-A signal and the eye’s resilience to structural stress. Because these hemodynamic variables were not comprehensively quantified in the present cohort, PeriVD should be interpreted as an integrated marker of local vulnerability rather than as a standalone causal mechanism.

Our strong association between drusen burden (height) and progression is also concordant with OCT-based anatomic studies that quantify drusen size/extent and relate these measures to damage. En face OCT/OCT-A work on buried ODD has shown that larger drusen are associated with characteristic perfusion defects (e.g., peripapillary capillary-layer vessel density decreases), supporting a mechanistic link between lesion size and vascular compromise [21]. Likewise, volumetric swept-source OCT quantification demonstrated a tight relationship between total drusen volume and VF loss (with MD worsening on the order of ~20 dB per mm^3^ increase in volume in a small series), implying that the “dose” of drusen material matters for function [22]. Our mediation findings are particularly compatible with this body of work: a substantial fraction of the drusen-height effect on 2-year MD deterioration was statistically mediated through peripapillary VD, suggesting that the burden–damage pathway is not purely mechanical, but partially expressed through microvascular compromise. In practical terms, this supports multimodal monitoring that pairs drusen metrics (height/segmentation where available) with perfusion measures to better represent both compressive load and vascular reserve.

Our mediation analysis also warrants cautious interpretation. The finding that 27.8% of the drusen height effect on 2-year MD deterioration was mediated through PeriVD supports biological plausibility, but it does not establish temporal directionality or direct causation. One plausible pathway is that enlarging or more anterior drusen mechanically compress the prelaminar microvasculature, reducing perfusion and accelerating axonal injury; another is that pre-existing microvascular insufficiency lowers tissue tolerance and amplifies the functional consequences of a given drusen burden. The most likely model is bidirectional interaction, and prospective imaging with longer serial follow-up will be required to determine whether vascular compromise precedes, parallels, or follows structural enlargement [12,14,18,19,20,21].

Finally, placing our longitudinal slopes in context, Estrela et al. quantified rates of VF change in eyes with ODD, reinforcing that progression is heterogeneous: many eyes decline slowly, while a clinically important subset deteriorates faster and benefits most from early identification and closer surveillance [23]. Our operational definition of “fast progression” (≤−0.5 dB/year) yielded a clearly separable phenotype (older age, worse baseline MD, lower peripapillary VD, higher drusen height, and greater 2-year RNFL loss), mirroring the broader literature’s emphasis on heterogeneity and the need for individualized monitoring intervals. The clinical implication is straightforward: combining (i) baseline functional status, (ii) objective neuroaxonal tissue metrics, (iii) drusen burden, and (iv) OCT-A perfusion provides a more complete representation of risk than any single domain alone, supporting earlier VF trend analysis (MD slope) and the prioritization of OCT-A in patients with large/superficial drusen or borderline structural thinning where the next clinical decision hinges on whether the eye is likely to be a rapid progressor.

These results support a pragmatic, clinic-ready stratification pathway for ODD: quantify the maximum drusen height on EDI-OCT and pair it with peripapillary OCT-A vessel density, then trend VF MD over time (MD slope) rather than relying on a single VF snapshot. Eyes with superficial ODD, large drusen, and reduced PeriVD concentrate risk (fast progression rates exceeding 50% in the lowest PeriVD tertile) suggest that they merit tighter follow-up intervals, earlier VF trend-based decisions, and more frequent structural monitoring (RNFL/GCC and contrast sensitivity), while eyes with preserved PeriVD and smaller drusen can often be monitored less intensively.

From a clinical decision-making standpoint, our results support a risk-enrichment range rather than a fixed universal cutoff. In descriptive terms, risk became more concentrated once the maximum drusen height approached approximately 350–380 µm, especially when PeriVD was concurrently reduced, but this range should be viewed as hypothesis-generating rather than as a validated threshold for management decisions. External validation with longer follow-up will be necessary before any single drusen-height cutoff can be recommended for routine use. Nevertheless, these findings should be considered in the study context, as different patient factors, demographics, and comorbidities may influence the applicability of these findings [24,25,26,27,28,29,30].

### 4.2. Study Limitations

This study has several limitations. First, it was a single-center cohort with retrospective extraction of imaging/perimetry metrics, which introduces potential selection bias, residual confounding, and limited transportability to other populations or imaging platforms. Second, although the minimum follow-up of 18 months was sufficient for estimating an annualized MD slope and identifying a clinically meaningful subgroup of fast progressors, ODD is typically a slowly progressive disorder, and longer observation windows (ideally 5–10 years) would provide a more complete view of disease trajectory. Third, OCT-A vessel density is device- and quality-dependent; excluding scans with motion artifacts, segmentation failure, or low signal strength created a technically cleaner dataset than routine clinical practice and may therefore overestimate real-world applicability. Fourth, maximum drusen height was measured manually, and formal inter-observer reproducibility statistics were not available in this retrospective dataset. Fifth, one eye per patient was analyzed and the study eye was defined by baseline MD (or drusen height when MD was similar), which may incompletely represent bilateral asymmetry. Finally, MD slope was modeled linearly for interpretability, but individual eyes may follow non-linear trajectories; prospective multicenter cohorts with denser serial testing are needed for external validation, the assessment of non-linear progression, and refinement of clinically actionable thresholds.

## 5. Conclusions

In adults with OCT-confirmed ODD, greater drusen height and reduced peripapillary vessel density jointly characterize a high-risk phenotype with faster VF decline. Superficial ODD, higher drusen burden, and lower PeriVD independently predict fast progression, and OCT-A adds meaningful incremental predictive value beyond clinical and OCT measures, supporting multimodal monitoring focused on structure, perfusion reserve, and longitudinal VF slope. These findings should be interpreted as a framework for risk enrichment rather than as a definitive externally validated risk score.

## Figures and Tables

**Table 1:** Demographic and systemic characteristics by drusen type (buried vs. superficial).

Variable	Buried ODD (*n* = 75)	Superficial ODD (*n* = 56)	*p*-Value
Age, years	39.9 ± 13.3	44.4 ± 11.1	0.037
Female sex	43 (57.3%)	38 (67.9%)	0.296
Arterial hypertension	24 (32.0%)	17 (30.4%)	0.992
Migraine	22 (29.3%)	27 (48.2%)	0.038
Hyperlipidemia	37 (49.3%)	24 (42.9%)	0.588
Obstructive sleep apnea	10 (13.3%)	8 (14.3%)	1
Current smoking	20 (26.7%)	12 (21.4%)	0.627
Spherical equivalent, D	1.1 ± 1.6	0.9 ± 1.7	0.475
Axial length, mm	22.6 ± 0.8	22.9 ± 0.9	0.033

ODD, optic disc drusen; D, diopters; mm, millimeters. Data are mean ± SD or *n* (%). *p*-values from between-group comparisons (*t* test for continuous variables; χ^2^/Fisher’s exact test for categorical variables, as appropriate).

**Table 2:** Multimodal structural and microvascular imaging features by drusen type.

Variable	Buried ODD (*n* = 75)	Superficial ODD (*n* = 56)	*p*-Value
Maximum drusen height, µm	247.2 ± 92.8	382.6 ± 110.9	<0.001
PHOMS present	49 (65.3%)	33 (58.9%)	0.564
Bilateral ODD	47 (62.7%)	33 (58.9%)	0.786
Global RNFL, µm	99.9 ± 9.4	96.4 ± 11.9	0.077
Global GCC, µm	93.0 ± 7.2	91.4 ± 7.9	0.216
Peripapillary vessel density, %	50.6 ± 2.6	50.0 ± 2.7	0.255
Intra-disc vessel density, %	44.4 ± 2.8	43.3 ± 2.3	0.019
Any reproducible VF defect	44 (58.7%)	44 (78.6%)	0.02

ODD, optic disc drusen; PHOMS, peripapillary hyperreflective ovoid mass-like structures; RNFL, retinal nerve fiber layer; GCC, ganglion cell complex; VF, visual field; µm, micrometers. Data are mean ± SD or *n* (%).

**Table 3:** Structure–function and progression across peripapillary vessel density tertiles.

Variable	Low VD (*n* = 44)	Mid VD (*n* = 43)	High VD (*n* = 44)	*p*-Value
Peripapillary vessel density, %	47.3 ± 1.7	50.3 ± 0.6	52.9 ± 1.4	<0.001
Global RNFL, µm	93.4 ± 10.0	99.3 ± 9.7	100.4 ± 11.9	0.005
Global GCC, µm	90.6 ± 5.6	92.4 ± 8.5	94.1 ± 7.4	0.043
Visual field MD, dB	−4.9 ± 2.5	−4.1 ± 2.5	−3.8 ± 2.8	0.093
Contrast sensitivity (logCS)	1.4 ± 0.2	1.6 ± 0.2	1.6 ± 0.2	<0.001
Maximum drusen height, µm	333.4 ± 127.9	285.6 ± 113.6	252.9 ± 114.2	0.01
MD slope, dB/year	−0.4 ± 0.2	−0.3 ± 0.2	−0.2 ± 0.2	<0.001
Fast progression (≤−0.5 dB/year)	23 (52.3%)	7 (16.3%)	6 (13.6%)	<0.001
Any reproducible VF defect	35 (79.5%)	28 (65.1%)	25 (56.8%)	0.071
Transient visual obscurations	14 (31.8%)	25 (58.1%)	16 (36.4%)	0.03

VD, vessel density; RNFL, retinal nerve fiber layer; GCC, ganglion cell complex; VF, visual field; MD, mean deviation; dB, decibels; logCS, log contrast sensitivity; µm, micrometers. Data are mean ± SD or *n* (%).

**Table 4:** Baseline phenotype and 2-year outcomes in fast progressors vs. slow/stable ODD.

Variable	Slow/Stable (*n* = 95)	Fast Progressors (*n* = 36)	*p*-Value
Age, years	39.9 ± 12.3	47.1 ± 11.8	0.003
Superficial ODD	29 (30.5%)	27 (75.0%)	<0.001
Migraine	33 (34.7%)	16 (44.4%)	0.411
Peripapillary vessel density, %	50.7 ± 2.4	48.8 ± 2.8	<0.001
Maximum drusen height, µm	258.5 ± 109.9	375.6 ± 114.4	<0.001
Global RNFL, µm	99.3 ± 10.1	93.4 ± 11.9	0.01
Contrast sensitivity (logCS)	1.6 ± 0.2	1.4 ± 0.2	<0.001
Baseline visual field MD, dB	−3.9 ± 2.8	−5.1 ± 2.1	0.009
MD slope, dB/year	−0.2 ± 0.2	−0.6 ± 0.1	<0.001
RNFL loss over 2 years, µm	1.6 ± 0.9	2.4 ± 0.8	<0.001
MD deterioration over 2 years, dB	0.6 ± 0.5	1.2 ± 0.4	<0.001
Any reproducible VF defect	58 (61.1%)	30 (83.3%)	0.027

ODD, optic disc drusen; RNFL, retinal nerve fiber layer; VF, visual field; MD, mean deviation; dB, decibels; logCS, log contrast sensitivity; µm, micrometers. Data are mean ± SD or *n* (%).

**Table 5:** Correlations of baseline MD and MD slope with structural and microvascular metrics (Pearson r).

Predictor	r with Baseline MD	*p*-Value	r with MD Slope	*p*-Value
DrusenHeight	−0.2	0.026	−0.5	<0.001
PeriVD	0.2	0.012	0.4	<0.001
DiscVD	0.1	0.327	0.3	0.003
RNFL	0.1	0.559	0.3	<0.001
GCC	0.2	0.013	0.3	0.001
Age	−0.3	<0.001	−0.3	0.001
LogCS	0.3	<0.001	0.4	<0.001
BCVA	−0.2	0.063	−0.2	0.014

MD, mean deviation; RNFL, retinal nerve fiber layer; GCC, ganglion cell complex; PeriVD, peripapillary vessel density; DiscVD, intra-disc vessel density; logCS, log contrast sensitivity; BCVA, best-corrected visual acuity; r, Pearson correlation coefficient.

**Table 6:** Multivariable logistic regression predicting fast progression (MD slope ≤ −0.5 dB/year).

Predictor	Adjusted OR	95% CI	*p*-Value
Age (per 10 years)	1.3	0.8–2.1	0.383
Drusen height (per 100 µm)	2	1.3–3.2	0.003
Peripapillary VD (per 1%)	0.8	0.6–1.0	0.046
Baseline MD (per 1 dB)	0.9	0.7–1.1	0.18
Migraine (yes vs. no)	0.6	0.2–1.7	0.326
Hypertension (yes vs. no)	0.8	0.3–2.2	0.637
Superficial vs. buried	6.3	2.1–18.5	<0.001

MD, mean deviation; dB, decibels; OR, odds ratio; CI, confidence interval; VD, vessel density; µm, micrometers.

**Table 7:** Does peripapillary vessel density partly mediate the effect of drusen height on 2-year visual field deterioration?

Effect Estimate (per +100.0 µm Drusen Height)	Effect on 2-Year MD Deterioration (dB)	95% CI	*p*-Value
Total effect	0.3	0.2 to 0.5	<0.001
Natural direct effect (not through VD)	0.2	0.1 to 0.4	0.002
Natural indirect effect (through VD)	0.1	0.0 to 0.2	0.017
Proportion mediated (%)	27.8	11.6 to 46.9	0.004

Exposure: Drusen height (per 100.0 µm). Mediator: Peripapillary VD (%, continuous). Outcome: 2-year MD deterioration (dB) (larger = worse). Method: Counterfactual mediation with 5000 bootstrap resamples for CI and *p*-values. Sensitivity note (robustness): the indirect effect remained significant after additionally adjusting for baseline RNFL (*p* = 0.031). VD, vessel density; MD, mean deviation; dB, decibels; CI, confidence interval; µm, micrometers.

**Table 8:** Incremental clinical utility of adding OCT-A to a progression-risk model.

Metric	Model A	Model B	Δ (B − A)	*p*-Value
AUC	0.8	0.9	0.1	0.011
Brier score	0.2	0.1	−0.1	0.019
Calibration slope	0.9	1	0.1	0.044
Calibration-in-the-large	0.1	0	−0.1	0.062
Categorical NRI (%)	—	—	22.6	0.008
IDI (%)	—	—	6.4	0.014
Likelihood ratio test	—	—	—	0.003

Outcome: Fast progression (MD slope ≤ −0.5 dB/year). Risk categories for NRI: <15%, 15–35%, >35%. Comparison: Model A (Clinical + OCT): age, baseline MD, RNFL, drusen height, superficial/buried; Model B (Model A + OCT-A): adds peripapillary VD and intra-disc VD. Direction of reclassification (for NRI detail): among fast progressors, 13/36 (36.1%) moved to a higher risk category and 3/36 (8.3%) moved down; among slow/stable, 10/95 (10.5%) moved down and 6/95 (6.3%) moved up. OCT-A, optical coherence tomography angiography; OCT, optical coherence tomography; AUC, area under the curve; NRI, net reclassification improvement; IDI, integrated discrimination improvement.

## Data Availability

The data presented in this study are available on request from the corresponding author due to ethical reasons.
